# Transcriptional Regulation of the *HMGA1* Gene by Octamer-Binding Proteins Oct-1 and Oct-2

**DOI:** 10.1371/journal.pone.0083969

**Published:** 2013-12-18

**Authors:** Eusebio Chiefari, Biagio Arcidiacono, Katiuscia Possidente, Stefania Iiritano, Valeria Ventura, Rosantony Pandolfo, Francesco Saverio Brunetti, Manfredi Greco, Daniela Foti, Antonio Brunetti

**Affiliations:** 1 Department of Health Sciences, University “Magna Græcia” of Catanzaro, Viale Europa (Loc. Germaneto), Catanzaro, Italy; 2 Department of Medical and Surgical Sciences, University “Magna Græcia” of Catanzaro, Viale Europa (Loc. Germaneto), Catanzaro, Italy; 3 Department of Experimental and Clinical Medicine, University “Magna Græcia” of Catanzaro, Viale Europa (Loc. Germaneto), Catanzaro, Italy; Indian Institute of Science, India

## Abstract

The High-Mobility Group AT-Hook 1 (HMGA1) protein is an architectural transcription factor that binds to AT-rich sequences in the promoter region of DNA and functions as a specific cofactor for gene activation. Previously, we demonstrated that HMGA1 is a key regulator of the insulin receptor (*INSR*) gene and an important downstream target of the INSR signaling cascade. Moreover, from a pathogenic point of view, overexpression of HMGA1 has been associated with human cancer, whereas functional variants of the *HMGA1* gene have been recently linked to type 2 diabetes mellitus and metabolic syndrome. However, despite of this biological and pathological relevance, the mechanisms that control *HMGA1* gene expression remain unknown. In this study, to define the molecular mechanism(s) that regulate *HMGA1* gene expression, the *HMGA1* gene promoter was investigated by transient transfection of different cell lines, either before or after DNA and siRNA cotransfections. An octamer motif was identified as an important element of transcriptional regulation of this gene, the interaction of which with the octamer transcription factors Oct-1 and Oct-2 is crucial in modulating *HMGA1* gene and protein expression. Additionally, we demonstrate that HMGA1 binds its own promoter and contributes to its transactivation by Oct-2 (but not Oct-1), supporting its role in an auto-regulatory circuit. Overall, our results provide insight into the transcriptional regulation of the *HMGA1* gene, revealing a differential control exerted by both Oct-1 and Oct-2. Furthermore, they consistently support the hypothesis that a putative defect in Oct-1 and/or Oct-2, by affecting HMGA1 expression, may cause INSR dysfunction, leading to defects of the INSR signaling pathway.

## Introduction

HMGA1 is an architectural transcription factor that specifically interacts with the narrow minor groove of AT-rich regions of DNA and regulate gene expression [1], [2], [3]. By modifying DNA conformation and by recruiting transcription factors to the transcription start site, it facilitates the assembly and stability of stereospecific DNA-protein complexes (so-called enhanceosomes), thus driving gene transcription in response to multiple extracellular and intracellular signals [4], [5], [6]. In addition to its role in gene transcription, HMGA1 is also involved in numerous other processes, including embryogenesis, differentiation, and neoplastic transformation [7], [8], [9]. Recent evidence assigns to HMGA1 an important role in the transcriptional regulation of glucose homeostasis [10], [11], [12], [13], [14]. Consistent with these latter findings, we have shown that defects in HMGA1 expression and/or function, by negatively affecting insulin receptor (INSR) signaling in insulin target tissues, may cause insulin resistance and increase susceptibility to type 2 diabetes mellitus [11], [15]. Also, evidence has been provided implicating the *HMGA1* locus as one conferring a high cross-race risk of development of type 2 diabetes and metabolic syndrome [16], [17], [18], [19]. However, despite this variety of studies, the mechanisms that regulate *HMGA1* gene expression are mostly unknown.

Cloning and characterization of the *HMGA1* gene reveals a very complex structure [2], [20], with a regulatory region comprising the untranslated exons 1–3, lacking TATA and CAAT, but rich in G and C. Its complexity includes at least two transcription start sites and an extensive alternative mRNA splicing, particularly in the 5′-untranslated region [2], [20], resembling the genes that are regulated without the preferential utilization of any specific start site [21]. Instead, a preferential utilization of start site 2 has been demonstrated under certain experimental conditions, in which tight gene regulation is achieved, following the production of specific mRNA in response to different stimuli [20]. A mechanism of transcriptional regulation of the human *HMGA1* gene has been postulated before both in embryonic and cancer cells highly expressing HMGA1 [22]. It has been proposed that elevated expression of *HMGA1* in these cell models is mainly controlled by specificity protein 1 (Sp1) and activator protein-1 (AP-1) transcription factors, both of which seem to act as positive regulating elements, after binding to the transcription start site 1 and the transcription start site 2, respectively [22]. In the current study, we sought to further investigate the mechanisms that underlie *HMGA1* gene expression in different human and rat cell lines widely used in biochemical and molecular genetic studies of gene function and regulation. We report herein the identification of a new mechanism of regulation of the *HMGA1* gene, which involves the octamer DNA motif “ATGCAAAT” originally described in immunoglobulin (*Ig*) gene regulation [23]. As a *cis*-acting transcriptional regulatory element found in both promoters and enhancers of a wide variety of ubiquitously expressed and cell type-specific genes, this octamer motif is specifically bound by a family of at least 11 distinct nuclear proteins, named octamer-binding (Oct) transcription factors, designated from Oct-1 to Oct-11 [24]. All these factors are characterized by two Pit-Oct-Unc (POU) domains, in which the N-terminal subunit is known as the POU-specific (POUS) domain, while the C-terminal subunit is a homeo domain (POUH). Both domains are required for high-affinity, site-specific binding to the octamer motif, and mediate specific protein-protein interactions with other transcription factors or co-factors, thus activating or inhibiting gene expression [24]. Here, we focused our attention on the regulation of *HMGA1* gene transcription by the octamer-binding proteins Oct-1 and Oct-2, whose ubiquitous expression differ from other Oct proteins that exhibit, instead, a developmental and tissue-specific distribution [24]. Our data indicate a role for Oct-1 and Oct-2 in regulating HMGA1 expression through direct interaction with the upstream *HMGA1* promoter region.

## Materials and Methods

### Cells, protein extracts and Western Blot (WB)

HepG2 human hepatoma cells, and human cervix epithelioid carcinoma cells (HeLa) were maintained in DMEM (Invitrogen) supplemented with 10% fetal bovine serum (FBS). IM-9 cultured human lymphocytes (ATCC) were maintained at subconfluent densities in RPMI 1640 medium supplemented with 10% FBS, 2 mM glutamine, penicillin (100 U/ml), and streptomycin (100 µg/ml) in a humidified 5% CO_2_ atmosphere at 37°C. L6 muscle rat cells were differentiated in DMEM containing 2% FBS, penicillin (100 U/ml), streptomycin (100 µg/ml), and 2 mM glutamine in a humidified 5% CO_2_ atmosphere at 37°C. The medium was replaced every 3 days until myotube formation was reached. Total cellular lysates or nuclear extracts were prepared as previously [25] and final protein concentrations in the extracts were determined using the colorimetric assay of Bradford (Bio-Rad). WB analysis was performed on total cellular lysates or nuclear extracts as previously described [26]. The antibodies used for these studies were: anti-HMGA1 [26], anti-Oct-1 and anti-Oct-2 (Santa Cruz Biotech).

### Probes and electrophoresis mobility shift assay (EMSA)

**Figure 1:**
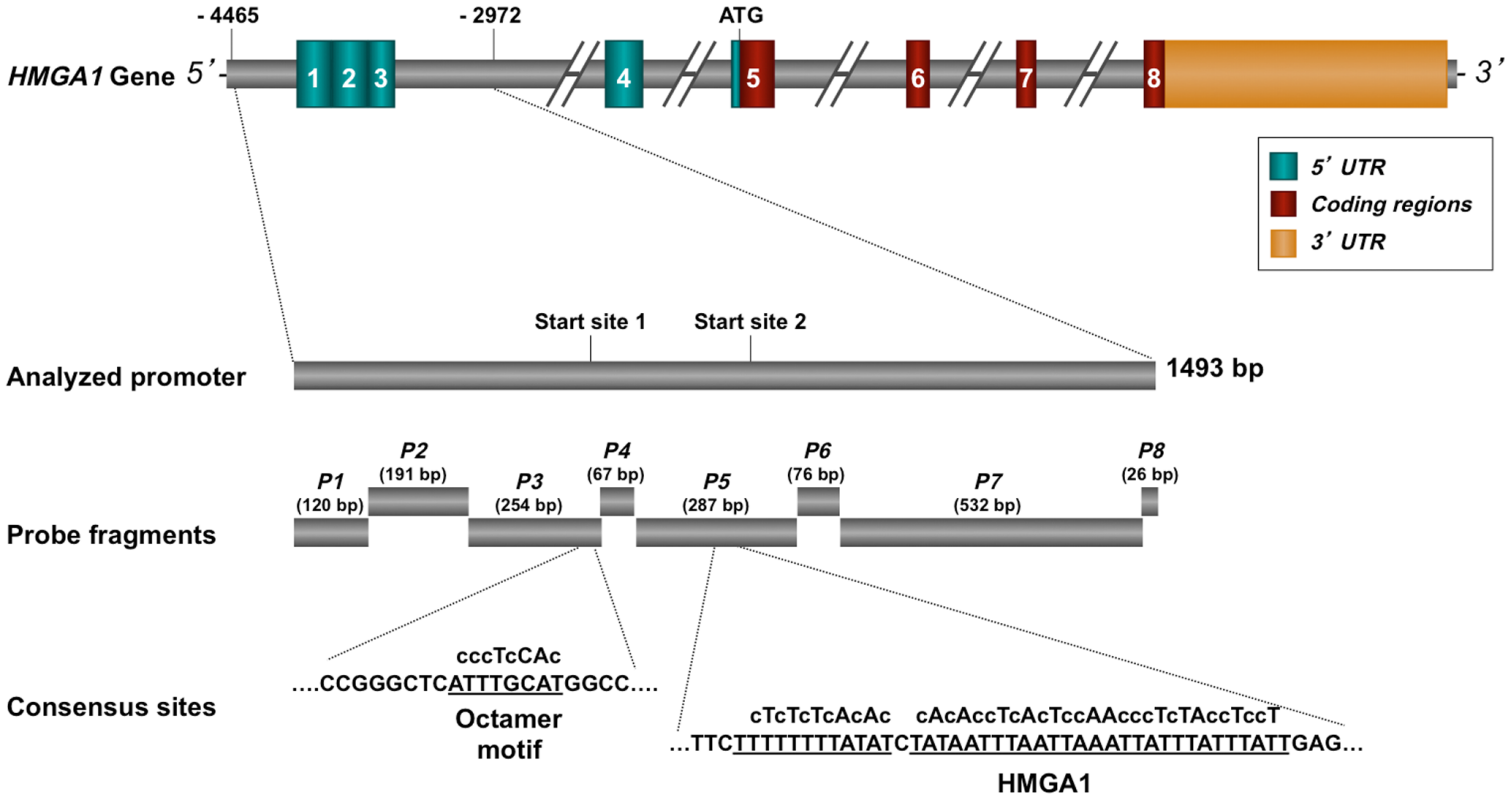
Schematic representation of the human *HMGA1* gene showing the eight fragments of the analyzed promoter region (*P1*-*P8*) used as probes in EMSAs. The octamer motif “ATTTGCAT” within fragment *P3* and the binding sequence for HMGA1 within fragment *P5* are underlined. Mutagenized bases in each consensus site are in lowercase letters.

To obtain probe DNA for EMSA, the regulatory region of the *HMGA1* gene was amplified by PCR reaction, using the following specific oligonucleotide primers for the *HMGA1* gene (GenBank AL354740): *HMGA1* forward 1 5′-TTGTATCTCACCAATCAGCC-3′ (sense primer) and *HMGA1* reverse 1 5′-TCACGAAGGAGCTTCTGGCGG-3′ (antisense primer); *HMGA1* forward 2 5′-TGTACCAGGGAGGAAGGATACC-3′ (sense primer) and *HMGA1* reverse 2 5′-CTCCCTCACTTCCAGAACTTC-3′ (antisense primer). The obtained fragments (311 and 1242 bp, respectively) were purified using the Qiagen Gel Extraction Kit (QIAGEN), and an aliquot of recovered DNA was sequenced by automatic genetic analyzer and compared to the published sequence (Genbank n. NC_000006.11). Then, both genomic sequences for *HMGA1* were digested into eight smaller fragments (P1 to P8, see Figure 1) with *EcoRI* and *DdeI* restriction endonucleases (Promega), end-labeled them with (α-^32^P)dATP (GE Healthcare) using DNA Polymerase I (Promega), and electrophoresed on 8% non-denaturing polyacrylamide gel. Double-stranded probes were excised from the gel, purified according to a standard protocol [27] and used in EMSA, as previously described [25], [26]. Briefly, cell nuclear extract or either Oct-2 enriched nuclear extract (Roche) or pure HMGA1 [26] were incubated with 2 ng of radiolabeled probe, in the presence of poly(dI-dC), which was used as competitor DNA, in a buffer containing 15 mM Hepes pH 7.9, 1 mM EDTA, 5% glycerol, 40 mM KCl and 0.5 mM DTT. After 30 min of incubation at 20°C, the reaction products were separated by electrophoresis through a 6% non-denaturing polyacrylamide gel in 0.5× TBE (1× TBE  = 50 mM Tris, 50 mM boric acid and 1 mM EDTA), and free and bound DNA were detected by autoradiography [25], [26]. Unlabeled 22-mer double stranded oligonucleotides containing consensus binding site for the transcription factors AP-1, AP-2, AP-3, Sp1, Oct-1, Oct-2 (Promega) and N-Myc (Santa Cruz Biotech) were used in competition studies, together with a 27-mer double-stranded oligonucleotide containing the PRDII element of the β-interferon promoter which specifically binds HMGA1 [4], [26], [28].

### Chromatin immunoprecipitation (ChIP)

ChIP was performed in HepG2 cells, either untreated or pretreated with *Oct-1* or *Oct-2* small interfering RNA (siRNA), as described previously [13]. Formaldehyde-fixed DNA-protein complex was immunoprecipitated with either anti-Oct-1 or Oct-2 antibody. Sequence-specific primers for the *HMGA1* gene promoter [human HMGA1 (NC_000006.11): *HMGA1* forward 5′-TGTACCAGGGAGGAAGGATAC-3′ and *HMGA1* reverse 5′-ATGGCGAGGGGCAGGAAGCTGG-3′] were used for PCR amplification of immunoprecipitated DNA (30 cycles), using PCR ready-to-go beads (GE Healthcare). PCR products were electrophoretically resolved on 1.5% agarose gel and visualized by ethidium bromide staining. Specifity of PCR product was confirmed by direct sequencing. Primers for quantitative reverse transcriptase PCR (qRT-PCR) of ChIP-ed samples are available upon request.

### Transfection studies

The *HMGA1-luciferase* (*HMGA1-luc*) reporter plasmid was obtained by cloning the *NheI*/*XhoI* 1427-bp sequence fragment of the human *HMGA1* gene promoter into pGL3-basic vector (Promega). Recombinant *HMGA1-luc* reporter construct, in the presence or absence of expression vectors for Oct-1 or Oct-2 [29], were transiently transfected into cultured cells using LipofectAMINE 2000 reagent (Invitrogen), and *luc* activity was assayed 48 hrs later, using the dual-luciferase reporter assay system (Promega) [30]. *Renilla* control vector served as an internal control of transfection efficiency, together with measurements of protein expression levels. siRNA targeted to human *HMGA1*, *Oct-1* or *Oct-2*, plus nonspecific siRNA controls with a similar GC content (Invitrogen) were transfected into cells at 50% to 60% confluency and cells were analyzed 48 to 96 hrs later, as described previously [31].

### Real-time PCR

For qRT-PCR, total cellular RNA was extracted from cells using the RNAqueous-4PCR kit and subjected to DNase treatment (Ambion). RNA levels were normalized against *18S* ribosomal RNA in each sample, and cDNAs were synthesized from 2 µg of total RNA using the RETROscript first strand synthesis kit (Ambion). Primers for human and rat *HMGA1*, *Oct-1*, *Oct-2*, *INSR*, *Sp1* and *RPS9* were designed according to sequences from the GenBank database. A real-time thermocycler (Eppendorf Mastercycler ep realplex ES) was used to perform quantitative PCR. SYBR Green fluorescence was measured, and relative quantification was made against the *RPS9* cDNA used as an internal standard [12]. All PCR reactions were done in triplicate.

### Statistical analysis

All calculations were performed with the SPSS 20.0 software (SPSS Inc.). Mean values were compared with *t*-tests. A p-value <0.05 (two-tailed) was considered significant.

## Results

### Putative binding sites on the *HMGA1* gene promoter

In an attempt to identify the mechanism(s) that regulate *HMGA1* gene expression, we initially performed functional experiments aimed at identifying protein-DNA interactions within the *P1* to *P8* sequence elements of the *HMGA1* gene promoter (Figure 1). Consensus transcription factor-binding sites within these eight regions were explored by using the TRANSFAC data-base (containing a large library of predefined matrix descriptions for known transcription factor recognition sequences) searched with MatInspector professional software [32]. Putative binding sites for the octamer-binding proteins Oct-1 and Oct-2, and HMGA1 were identified and characterized by EMSA using homologous radiolabeled fragments. As shown in Table 1, the octamer motif “ATGCAAAT” and the flanking sequence at both sides appeared highly conserved in evolution and located in the proximal promoter region of the *HMGA1* gene.

**Table 1:** Multiple sequence alignment of the *HMGA1* gene, including the octamer motif, from *Mus musculus, Bos Taurus, Pan troglodytes*, and *Homo sapiens*.

Species	Genbank	Sequences	Position
*Mus musculus*	NC_000083	ccccgggctc **atttgcat**gg ccccgccccc	−3020 from ATG
*Bos taurus*	NC_007324	ccccaggctc **atttgcat**gt ccccgccccc	−3668 from ATG
*Pan troglodytes*	NC_006473	ccccgggctc **atttgcat**gg ccccgccccc	−3979 from ATG
*Homo sapiens*	NC_000006.11	ccccgggctc **atttgcat**gg ccccgccccc	−3980 from ATG

The octamer sequence is underlined.

### Nuclear protein-DNA interaction within the *HMGA1* promoter region

**Figure 2:**
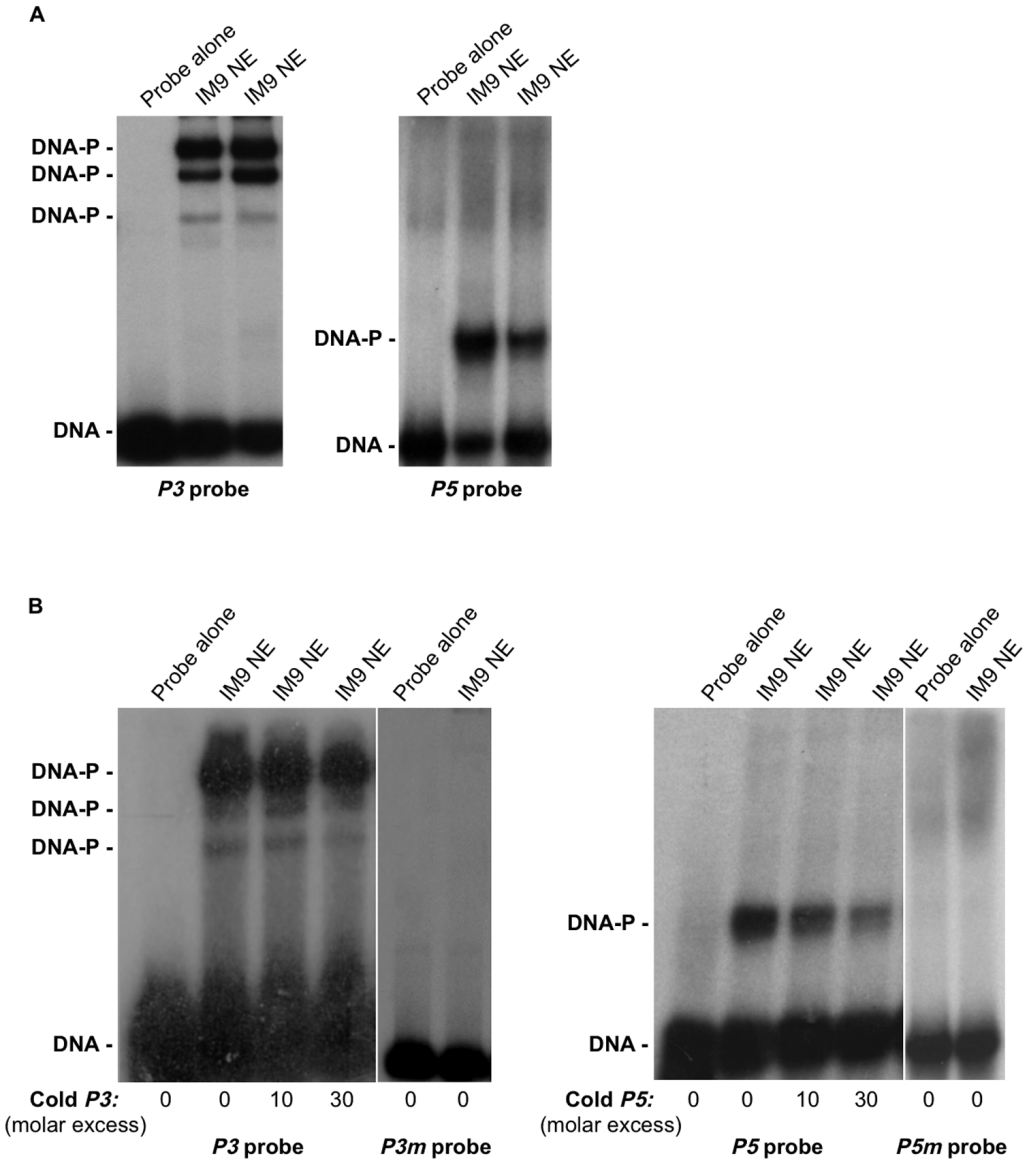
Nuclear protein-DNA interaction within *P3* and *P5* fragments of the *HMGA1* promoter. (A) Nuclear extracts (NE) from IM-9 cells were incubated with *P3* (left) or *P5* (right) probe (0.2 ng each), in the presence of 0.2 µg of poly(dI-dC), which was used as the competitor DNA for nonspecific DNA-binding proteins in the nuclear extracts. DNA protein complexes were resolved on a 6% non-denaturing polyacrylamide gel. (B) Labeled *P3* and *P5*, and a mutant version of either *P3* (*P3m*) or *P5* (*P5m*) probe were used in EMSAs with 4 µg of NE from IM-9 cells under the same conditions as in (A). Sequence specificity of HMGA1 DNA binding proteins was determined by using 10–30 fold molar excess of unlabeled fragment *P3* (left) or *P5* (right) as competitors. Free (DNA) and bound (DNA-P) probe is indicated. A representative of three separate assays is shown for each condition.

**Figure 3:**
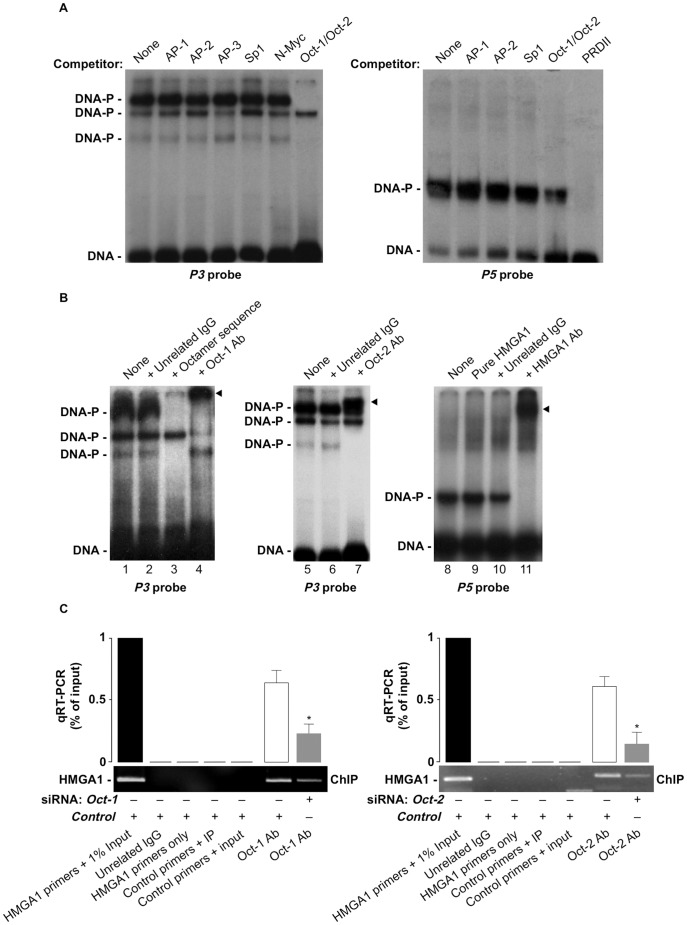
Identification of DNA-binding proteins. (A) Competitive EMSAs were performed with *P3* (left) and *P5* (right) probes in NE of IM-9 cells, in the absence (none) or presence of a 30-fold molar excess of consensus binding sites for the indicated transcription factors, or in the presence of either unlabeled competitor DNA for the octamer motif “ATGCAAAT” (lane Oct-1/Oct-2) or unlabeled PRDII oligonucleotide (lane PRDII). (B) EMSAs of radiolabeled fragments *P3* and *P5* with IM-9 NE (or 2.5 ng of pure HMGA1, lane 9). In supershift assays NE were preincubated with 1 µg of the polyclonal antibody (Ab) to Oct-1 (lane 4), Oct-2 (lane 7), or HMGA1 (lane 11), before addition of the probe. Supershifting of the Oct-1-, Oct-2- and HMGA1-DNA complexes are shown with an arrowhead. Control (unrelated rabbit serum IgG) antibody did not alter the mobility of the complexes (lanes 2, 6, 10). Lane 3, IM-9 NE plus an excess of unlabeled octamer motif. Free (DNA) and bound (DNA-P) probes are indicated. A representative of three separate assays is shown for each condition. (C) ChIP of the *HMGA1* gene promoter in IM-9 cells, untreated or pretreated with either *Oct-1* (left) or *Oct-2* (right) siRNA, using an anti-Oct-1 or anti-Oct-2 specific antibody (Ab), respectively. Representative assays are shown, together with qRT-PCR of ChIP-ed samples. *p<0.05 versus control (white bar).

As shown in Figure 2A, DNA-protein binding activity was observed following incubation of either probe *P3* or *P5* with nuclear extracts from IM-9 cells, a human B lymphocyte cell line expressing both Oct-1 and Oct-2 transcription factors, in addition to HMGA1. No binding activity was observed with any of the other six (*P1*, *P2*, *P4*, *P6*, *P7*, *P8*) DNA probes (not shown). To determine specificity of DNA-protein binding, competition assays were performed. A 10-30-fold molar excess of either unlabeled fragment *P3* or unlabeled fragment *P5* considerably reduced the binding of labeled *P3* and labeled *P5* to the DNA binding proteins, respectively (Figure 2B). Moreover, mutations within the consensus sequences abolished DNA-protein binding activity of either probe *P3* or *P5* (Figure 2B). As shown in Figure 3A, a 30-fold molar excess of 22-mer double stranded oligonucleotides representing the consensus binding sites for the nuclear factors AP-1, AP-2, AP-3, Sp1 and N-Myc did not compete for binding of nuclear proteins to *P3* and *P5* probes. Instead, unlabeled competitor DNA for the octamer motif “ATGCAAAT”, completely abolished protein-DNA complex formation with *P3* probe (Figure 3A), indicating that the POU domain is essential for the formation of a DNA-protein complex at this site. On the other hand, binding of HMGA1 to *P5* probe was displaced by excess unlabeled double-stranded oligonucleotides containing the *PRDII* element motif (GGGAAATTCCGTGGGAAATTCCGAGCT) (Figure 3A), which, as reported previously by us and others, specifically binds HMGA1 in the minor groove of the central AT-rich region [4], [26], [28], thus suggesting a potential autoregulatory role of HMGA1 on its own promoter. Next, we showed that the protein-DNA complexes formed upon incubation of nuclear extracts from IM-9 cells with labeled *P3* were recognized and supershifted to a slower-migrating form by the anti-Oct-1 or anti-Oct-2 antibody (Figure 3B); instead, binding of HMGA1 to labeled *P5* produced a protein-DNA complex that was recognized and supershifted by the anti-HMGA1 antibody (Figure 3B). Binding of Oct-1 and Oct-2 to *P3* sequence was substantiated further by ChIP coupled with qRT-PCR of ChIP-ed samples, showing that binding of both Oct-1 and Oct-2 to the endogenous *HMGA1* chromosomal locus was present in living IM-9 cells naturally expressing Oct-1 and Oct-2, and was considerably decreased in cells exposed to siRNA against either *Oct-1* or *Oct-2* (Figure 3C).

### HMGA1 is necessary for recruitment and binding of Oct-2 to the *HMGA1* promoter

**Figure 4:**
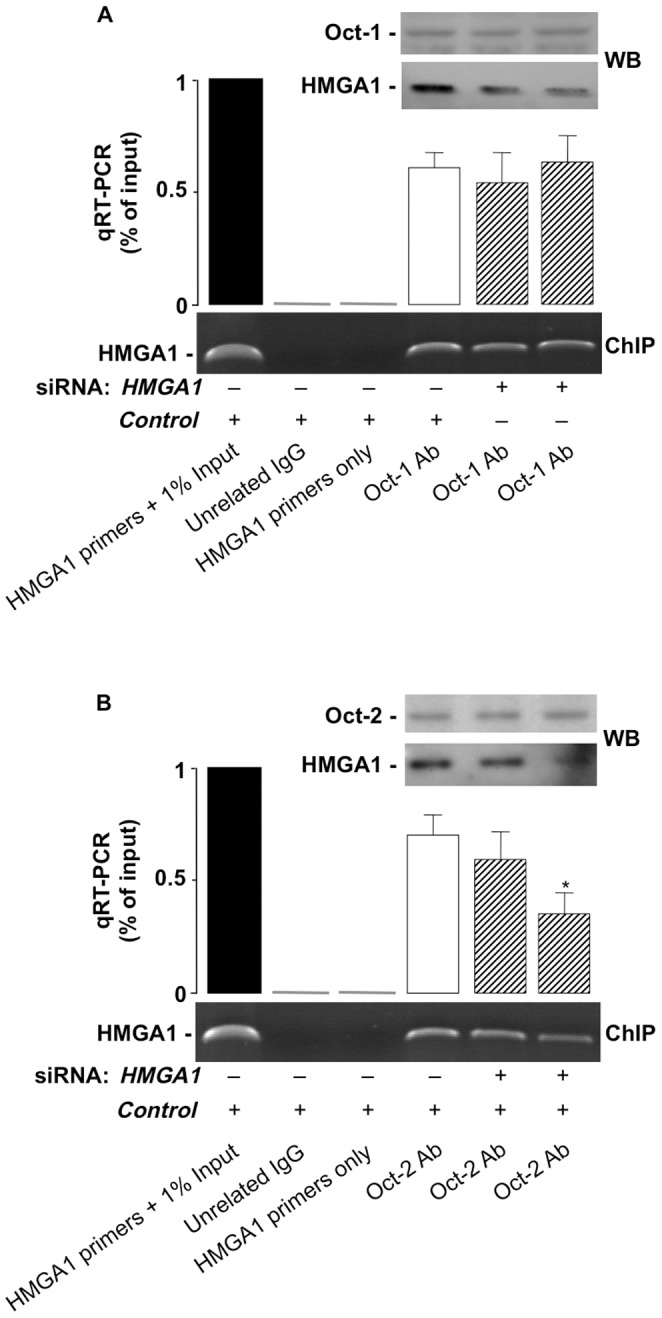
Effect of HMGA1 on the binding of Oct-1 and Oct-2 on the *HMGA1* gene promoter. (A,B) ChIP of the *HMGA1* promoter gene in IM-9 cells untreated or pretreated with *HMGA1* siRNA, using either an anti-Oct-1 or anti-Oct-2 specific antibody (Ab) as indicated. Representative assays are shown for each condition, together with qRT-PCR of ChIP-ed samples. *p<0.05 versus control (white bar). Western Blots (WB) of HMGA1, Oct-1, and Oct-2 in untreated and siRNA-treated cells are shown in the autoradiograms.

Physical association between HMGA1 and Oct-1 and Oct-2 has been reported previously in pull-down assays indicating that direct contact of Oct-1 and Oct-2 with HMGA1 occurs through their conserved POU homeodomains [33]. In the light of the above-mentioned data indicating that a relationship may exist between these factors, to see whether HMGA1 had any influence on the binding of Oct-1 and Oct-2 to the *HMGA1* promoter, we carried out ChIP experiments in living IM-9 cells, either untreated or pretreated with siRNA against *HMGA1*. As detected by qRT-PCR of ChIP-ed samples, whereas binding of Oct-1 to the *HMGA1* gene promoter was not affected in IM-9 cells expressing reduced levels of endogenous HMGA1 (Figure 4A), the binding of Oct-2 was significantly decreased in cells exposed to siRNA against *HMGA1* (Figure 4B), thus indicating that HMGA1 is required for binding of Oct-2, but not Oct-1, to the *HMGA1* gene promoter *in vivo*, in intact cells.

### Oct-1 and Oct-2 differentially regulate *HMGA1* gene promoter

**Figure 5:**
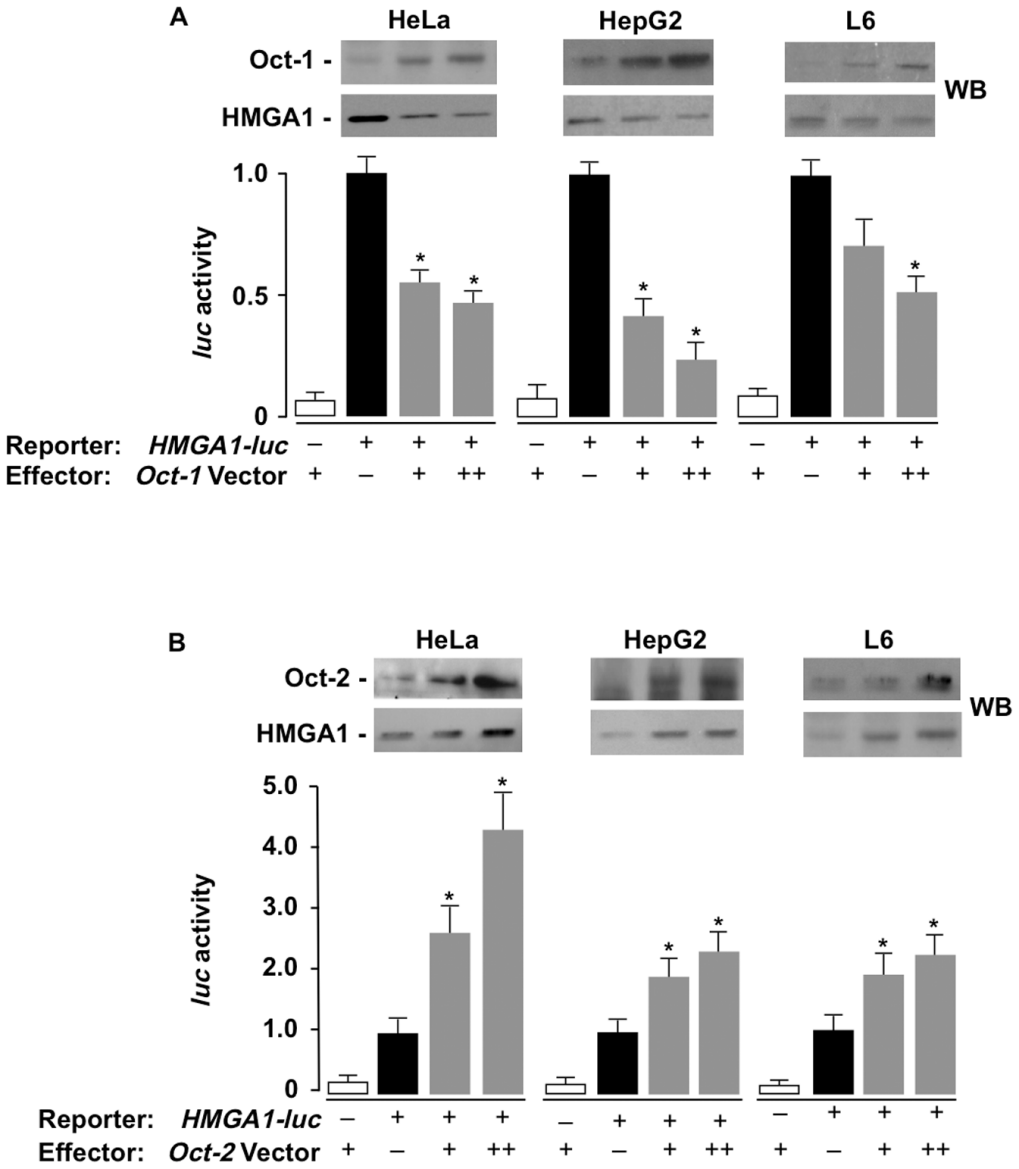
Functional significance of Oct-1 and Oct-2 for *HMGA1* gene transcription. (A,B) The recombinant human *HMGA1*-*luc* reporter plasmid (2.0 µg) was transfected into the indicated cell lines with increasing amounts (0, 1.0, 2.0 µg) of either an Oct-1 or Oct-2 expression vector. In either case, data represent the mean ± SEM for three separate experiments; values are expressed as factors by which *luc* activity decreased or increased as compared with the level of *luc* activity obtained in transfections with the reporter vector alone (black bars), which is assigned an arbitrary value of 1. White bar, pGL3-basic (vector without an insert). *p<0.05 versus control (black bar). Western Blots (WB) of HMGA1, Oct-1, and Oct-2 in each experimental condition are shown in the autoradiograms.

To test whether Oct-1 and Oct-2 had a functional role in regulating the *HMGA1* gene at the transcriptional level, human HeLa and HepG2 cells and rat skeletal muscle cells (differentiated from L6 myoblasts), three cell lines naturally expressing HMGA1, were cotransfected transiently with the *HMGA1*-*luc* reporter plasmid, plus increasing amounts of *Oct-1* or *Oct-2* expression vector. As shown in Figure 5A, overexpression of Oct-1 significantly reduced *HMGA1-luc* activity in all cell lines and this effect occurred in a dose-dependent manner. In contrast, overexpression of Oct-2 caused a significant increase of *HMGA1-luc* activity in all these cells (Figure 5B), supporting the notion that, by binding the same consensus site in the *HMGA1* promoter, Oct-1 and Oct-2 exert an opposite effect on the *HMGA1* gene: while the former decreases the transcriptional activity and protein level of HMGA1, the latter increases them.

### Oct-1 and Oct-2 may affect INSR expression

**Figure 6:**
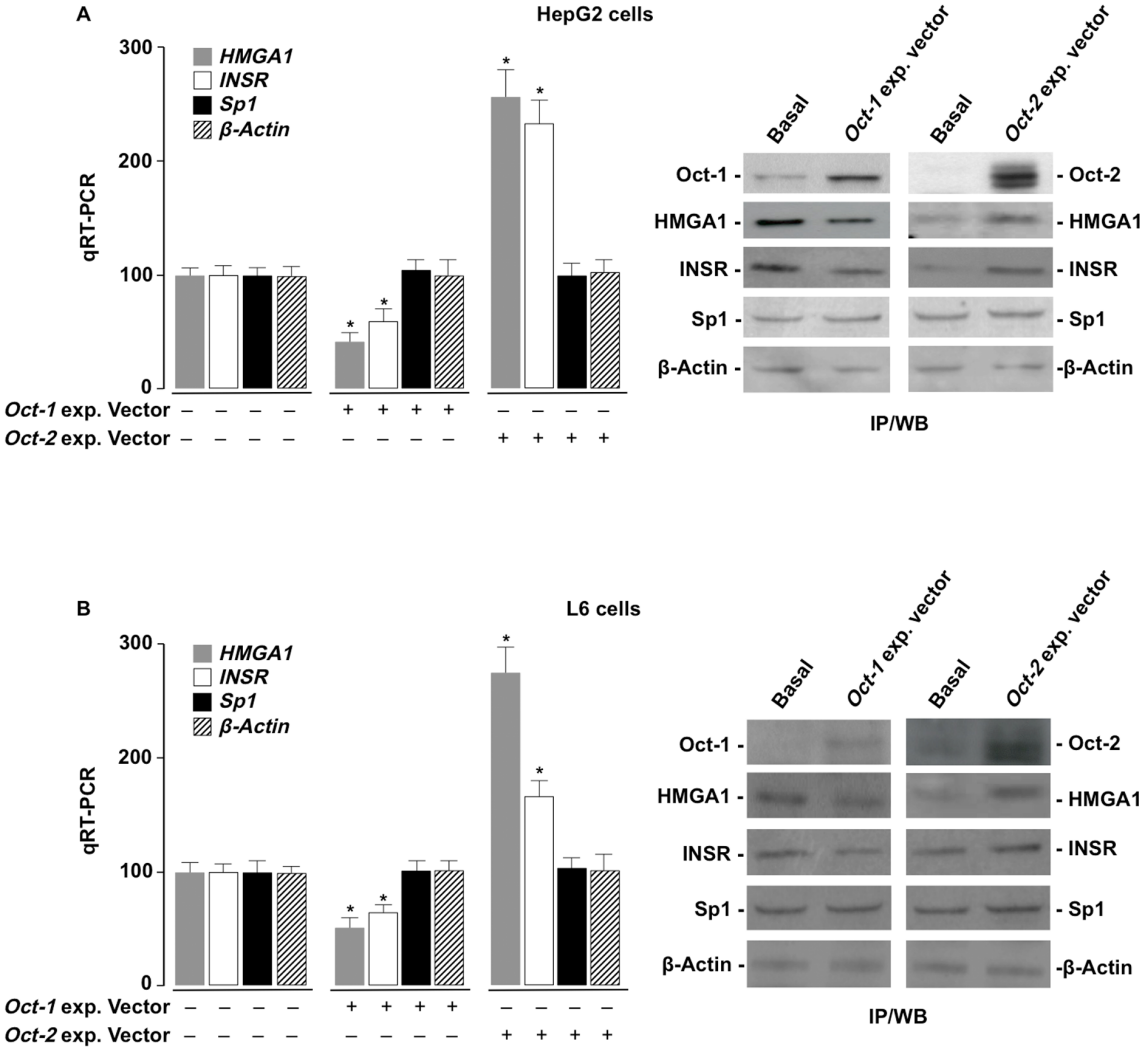
Role of Oct-1 and Oct-2 in HMGA1 expression and the consequent effects on INSR. (A,B) HepG2 and differentiated L6 cells were transfected with 2.0 µg of either Oct-1 or Oct-2 expression vector, and *HMGA1* and *INSR* mRNA (qRT-PCR) and protein (IP/WB) levels were measured. mRNA is expressed as percent of the value in untransfected (basal) HepG2 and L6 cells (100%). Results are the mean ± SEM of three independent experiments. *p<0.05 versus basal cells. Immunoprecipitation (IP) of the INSR and Western Blot (WB) were performed using an anti-INSR β-subunit antibody. Sp1 and β-actin, controls.

Based on the above data indicating that interaction of Oct-1 and Oct-2 with the *HMGA1* gene promoter may play a role in HMGA1 expression, it was important to ask whether this interaction could have an effect on the expression of the *INSR* gene, a well-characterized molecular target for HMGA1 which causes an increase in intracellular INSR in cells and tissues [10], [11], [26]. For this purpose, the ability of Oct-1 and Oct-2 to drive *INSR* gene expression was measured in HepG2 cells, in which protein levels of Oct-1 or Oct-2 were increased by transfecting cells with plasmids for expression of Oct-1 or Oct-2. As shown in Figure 6A, overexpression of *Oct-1* in HepG2 cells caused a decrease in endogenous *HMGA1* mRNA and protein levels and this was followed by a decline in *INSR* mRNA and protein expression. The opposite was observed when HepG2 cells were transfected with an expression vector coding for *Oct-2*: in this case, a significant increase was observed in the levels of HMGA1 mRNA and protein abundances, which were paralleled by increased levels of mRNA and protein for the INSR (Figure 6A). These effects were specific for HMGA1, as both mRNA and protein expression for the ubiquitously expressed transcription factor Sp1 were unaffected under both conditions. Similar results were obtained in differentiated L6 skeletal muscle cells naturally expressing INSR (Figure 6B). Thus, these data indicate that Oct-1 and Oct-2, by differentially modulating *HMGA1* gene transcription, may affect *INSR* gene and protein expression and perhaps the expression of other HMGA1-target genes.

## Discussion

For the first time in this work we identified and characterized a novel molecular mechanism that contribute to the regulation of *HMGA1* gene expression. Using DNA-protein interaction studies combined with ChIP coupled with qRT-PCR, we demonstrated a differential role for Oct-1 and Oct-2 transcription factors in the transcriptional regulation of the *HMGA1* gene which, in addition to the regulation of HMGA1 mRNA and protein expression, may contribute to the modulation of *HMGA1*-target genes. The observation that Oct-1 and Oct-2 differentially affect *HMGA1* gene expression by binding to the same octamer motif “ATGCAAAT” is consistent with previous reports, in which it has been demonstrated that both Oct-1 and Oct-2 bind to the same DNA sequence but may differ in their activation potential [29], [34], [35], [36]. Furthermore, here we show that, by facilitating the interaction of Oct-2 (but not Oct-1) with the *HMGA1* gene, HMGA1 enhances transactivation of its own promoter by this octamer transcription factor, providing evidence for the existence of an auto-regulatory circuit in which HMGA1 activates its own transcription. In relation to Oct-1, it needs to be considered that several isoforms of this transcription factor were described previously, each of which is active in a tissue-specific manner, being able to mediate different and even opposing effects [24]. Thus, we cannot exclude that in other cells the effect of Oct-1 on HMGA1 may be different from that reported here in our work. In this regard, results have been provided indicating that Oct-1, NF-kappa B and HMGA1 cooperate for transactivation of the *iNOS* promoter in pancreatic β-cells [37].

As we previously reported, HMGA1 is a main activator of the *INSR* gene [10], [26], and individuals with defects in HMGA1 have decreased INSR expression and increased susceptibility to type 2 diabetes mellitus [11], [15], [16], [18]. Also, we showed that, as a novel downstream nuclear target of the INSR signaling pathway [13], HMGA1 represents an important novel mediator of glucose disposal and glucose homeostasis [12], [14]. Therefore, based on these observations and the results from the present study indicating that a functional relation can be established between HMGA1, Oct-1 and Oct-2 in the context of the *INSR* gene, it is tempting to hypothesize that a putative defect in these nuclear proteins may cause INSR dysfunction with subsequent impairment of insulin signaling and action. In this regard, an association between *Oct-1* gene variants and type 2 diabetes mellitus, which may support this intriguing hypothesis, has been reported [38]. Moreover, recent evidences suggest that Oct-1 may play a role in metabolic homeostasis by acting as a sensor for cAMP (cyclic AMP). In particular, elevation of cAMP in pancreatic and intestinal endocrine cells reduces nuclear Oct-1 content [39], [40], and this is consistent with previous observations indicating that expression of HMGA1 increases in response to cAMP [12], [41], thus suggesting that increased HMGA1 expression could be due, at least in part, to the reduction of Oct-1 content.

In conclusion, our findings from this study reveal new mechanistic insight into the transcriptional mechanisms governing *HMGA1* gene expression. While additional investigations will be needed to further define the roles of Oct-1 and Oct-2 in HMGA1 expression in other cell systems and models, these results provide a paradigm to suggest other types of molecule, Oct-1 and Oct-2, in addition to HMGA1, which should be searched for in investigations designed to elucidate the causes of impairment of INSR signaling in humans with INSR dysfunction and insulin resistance.
